# Prevalence of characteristics associated with sarcopenia in elders: a cross-sectional study

**DOI:** 10.1590/0034-7167-2022-0209

**Published:** 2023-03-27

**Authors:** Caroline Ribeiro de Sousa, Janaína Fonseca Victor Coutinho, Marília Braga Marques, Rachel Gabriel Bastos Barbosa, Jarbas de Sá Roriz, Edson Silva Soares, Charlys Barbosa Nogueira, Rodrigo Lopes de Paula Souza

**Affiliations:** I Universidade Federal do Ceará. Fortaleza, Ceará. Brazil

**Keywords:** Nursing, Health of the Elderly, Sarcopenia, Primary Health Care, Public Health., Enfermería, Salud del Anciano, Sarcopenia, Atención Primaria de Salud, Salud Pública., Enfermagem, Saúde do Idoso, Sarcopenia, Atenção Primária à, Saúde, Saúde Pública.

## Abstract

**Objectives:**

to identify the prevalence and characteristics associated with sarcopenia in elders in Primary Health Care Units.

**Methods:**

cross-sectional study with 384 elders. To evaluate sarcopenia, we measured: strength and muscle mass, and physical performance. The elderly were classified as having: probable sarcopenia; sarcopenia; or severe sarcopenia. The chi-squared test and the multinomial logistic regression method were used.

**Results:**

the prevalence of probable sarcopenia was 25.52%; of sarcopenia, 11.98%; and of severe sarcopenia, 9.90%. Probable sarcopenia is 1.75 times more prevalent in men; osteoporosis is 2.16 times more prevalent in people with severe sarcopenia; polypharmacy is 1.57 times more likely in individuals with probable sarcopenia; and calf circumference below 31 cm is 2.24 times more likely in patients with sarcopenia and 2.19 times more likely in patients with severe sarcopenia.

**Conclusions:**

the highest prevalence was of probable sarcopenia, and the characteristics associated with sarcopenia were sex, osteoporosis, polypharmacy, overweight, obesity, and calf circumference.

## RESUMO

**Objetivos:**

identificar a prevalência e as características associadas à sarcopenia em pessoas idosas de Unidades de Atenção Primária à Saúde.

**Métodos:**

estudo transversal, com 384 pessoas idosas. Para avaliação de sarcopenia, mediu-se: força e massa muscular, desempenho físico. Classificaram se pessoas idosas com: sarcopenia provável; sarcopenia; e sarcopenia grave. Analisou-se com teste de qui quadrado e método de regressão logística multinomial.

**Resultados:**

a prevalência de provável sarcopenia foi de 25,52%; sarcopenia, 11,98%; e sarcopenia grave, 9,90%. Homens são 1,75 vez mais prevalentes em indivíduos com provável sarcopenia; osteoporose é 2,16 vezes mais prevalente na sarcopenia grave; polifarmácia, 1,57 vez mais prevalente na provável sarcopenia; circunferência da panturrilha menor que 31 cm é 2,24 vezes mais prevalente na sarcopenia e 2,19 vezes na sarcopenia grave.

**Conclusões:**

houve maior prevalência de provável sarcopenia, e as características associadas à sarcopenia foram: sexo, osteoporose, polifarmácia, sobrepeso, obesidade e circunferência da panturrilha.

## RESUMEN

**Objetivos:**

identificar prevalencia y características relacionadas a la sarcopenia en personas ancianas de Unidades de Atención Primaria de Salud.

**Métodos:**

estudio transversal, con 384 personas ancianas. Para evaluación de sarcopenia, medidos: fuerza y masa muscular, desempeño físico. Clasificadas personas ancianas con: sarcopenia probable; sarcopenia; y sarcopenia grave. Analizado con prueba chi cuadrado y método de regresión logística multinomial.

**Resultados:**

la prevalencia de probable sarcopenia fue de 25,52%; sarcopenia, 11,98%; y sarcopenia grave, 9,90%. Hombres son 1,75 vez más prevalentes en individuos con probable sarcopenia; osteoporosis es 2,16 veces más prevalente en la sarcopenia grave; polifarmacia, 1,57 vez más prevalente en la probable sarcopenia; circunferencia de los gemelos menor que 31 cm es 2,24 veces más prevalente en la sarcopenia y 2,19 veces en la sarcopenia grave.

**Conclusiones:**

Hubo mayor prevalencia de probable sarcopenia, y las características relacionadas a la sarcopenia fueron: sexo, osteoporosis, polifarmacia, sobrepeso, obesidad y circunferencia de los gemelos.

## INTRODUCTION

The loss of muscle mass has been studied for nearly 30 years, receiving the name of sarcopenia^(1)^. However, in 2016 it was recognized as a muscle disease (ICD-10-MC-M62.84), characterized by the loss of strength and muscle amount. Its causes are multifactorial and involve: aging, genetics, hormone and muscle tissue alterations, neurological decline, increased levels of pro-inflammatory cytokines, and mitochondrial dysfunctions^(2-4)^.

Worldwide, the prevalence of sarcopenia may vary from 3% to 86.5%^(5)^. In Brazil, its prevalence is of 15.4%, albeit with differences between cities. In Florianópolis, its prevalence is 33.3%; in São Paulo, 4.8%; in Salvador, 17.8%; and in Natal 10.7%^(6-9)^. This variation is due to ethnicity, place of residence (urban or rural), area researched (community, hospital, outpatient clinic, or long-permanence institution), instruments, methods, and cutoff points for diagnosis^(10-11)^.

Studies indicate that some characteristics, such as age, sex, level of physical activity, and the presence of chronic diseases are associated with the presence of sarcopenia^(3,10)^. Nonetheless, it is still necessary to understand its many possible causes^(12)^. Furthermore, when it is not treated, this condition has severe personal, social, and economic tolls, due to the fact it impairs daily-life activities and leads to lower functional capabilities, falls, fractures, institutionalization, hospitalization, and death^(13)^.

In this context, recognizing the issue and intervening as soon as possible leads to better outcomes in sarcopenia patients. Consequently, the importance of evaluations in Primary Health Care is evident, as this level of care is the one responsible for stratifying, screening, embracing, developing actions, and ensuring integral and continuous care to the elderly^(14)^.

Considering the above, increasing our understanding about this disease and raising awareness about its characteristics in different contexts is essential to develop diagnostic possibilities and interventions that can prevent it and promote health, which, in turn, will lead to better care and more quality of life for the elderly.

## OBJECTIVES

To identify the prevalence and characteristics associated with sarcopenia in the elderly of Primary Health Care Units.

## METHODS

### Ethical aspects

This study followed all ethical precepts. Its protocol was approved by the Research Ethics Committee in 2018.

### Design, period, and place of study

Cross-sectional epidemiological study. The guidelines of the EQUATOR network were followed through the use of the tool *Strengthening the Reporting of Observational Studies in Epidemiology* (STROBE)^(15)^. The study was carried out from April 2018 to June 2019, with elders being attended in six Primary Health Care Units (PHCUs) in Fortaleza, a city in the state of Ceará (CE).

### Population or sample; criteria for inclusion and exclusion

The study population was formed by 105,833 elders registered in the PHCUs in Fortaleza. The sample was calculated using the formula of cross-sectional studies with infinite populations, a population proportion of 50%, 5% error, and confidence interval of 95%, to a total of 384 elders.

Fortaleza is divided in six Regional Secretariats (RS), and we chose the metho d of stratified sampling due to the heterogeneous subdivision of elders among them. The PHCUs that attended to the highest number of elders in each secretariat were chosen, and the value from the sampling calculation was divided according with the percentage of elders registered in each, according to data made available by the Fortaleza Health Secretariat (SR I, 25%; SR II, 13%; SR III, 11%; SR IV, 9%; SR V, 27%; e SR VI, 15%).

Elders who went to the unit randomly were invited to participate in the research. Individuals aged 60 years or older who received attention in the PHCUs were included. Elders diagnosed with dementia according to their companions or to medical reports were excluded. 414 elders were recruited. 5 of them were excluded due to dementia diagnosis, and 25 instruments were not filled in properly by the researchers. As a result, the study counted on the participation of 384 people.

### Study protocol

Sociodemographic and clinical data were collected using a structured instrument in the form of a self-report. We investigated: age, sex, educational level, income in minimum wages (R$ 954.00 in 2018 and R$ 998.00 in 2019), retirement, marital status, housing, physical activity, number of falls in the last 12 months, drinking, smoking, comorbidities (hypertension, diabetes, cancer, osteoarthritis, cardiopathy, chronic kidney disease, osteoporosis, dyslipidemia, depression, anxiety, Parkinson’s disease, glaucoma, hypothyroidism, and schizophrenia

Anthropometric evaluations were carried out by measuring the weight, height, and the Body Mass Index (BMI), using the classification criteria determined by the Pan-American Health Organization^(16)^. The calf circumference was evaluated, with values below 31 cm being indicative of decrease in muscle mass.

Sarcopenia was evaluated using criteria from the *European Working Group on Sarcopenia in Older People 2* (EWGSOP2), which determine the following classification: probable sarcopenia, when the only symptom is low muscle strength; sarcopenia, when low muscle quantity/quality is confirmed; and severe sarcopenia, when it is possible to detect low muscle strength, low muscle quantity/quality, and low physical performance^(3)^.

The most commonly used method to measure physical performance is the measurement of gait speed in the 10-Meter Test^(17)^, whose cutoff point indicative of lower physical performance is 0.8 m/s^(3)^.

Grip strength was measured using a Jamar hydraulic dynamometer adjusted to level 2, a level in which the grip strength performance is the highest^(18)^. The cutoff points vary according with gender, with 27kgf for men and 16 kgf for women. Lower values indicate low muscle strength^(3)^.

Among methods available to evaluate muscle mass, we chose using the anthropometric equation to calculate Total Muscle Mass (TMM). This equation was developed, validated, and compare with body composition evaluation results, as calculated using *Dual Energy X-Ray Absorptiometry* (DEXA). The DEXA is considered to be the most recommended method. However, it is very costly and requires specialized professionals and equipment, meaning it is not accessible to all levels of health^(19)^.

The MMT (kg) is established using the formula: *TMM* = (0.244 × *body mass*) + (7.8 × *stature*) - (0.098 × *age*) + (6.6 × *sex*) + (*ethnicity* - 3.3). For the variable sex: 0 = women, 1 = men; for self-referred ethnicity, which as categorized later, the following values were adopted: 0=white (white, mixed, and native), -1,2 = Asian; and -1.4 = African ascent (black and brown)^(19)^. According with TMM, the Muscle Mass Index was calculated [MMI = TMM/stature^2^]. Later, it was classified according with the cutoff points proposed by European Consensus: men < 7.0 kg/m^2^, and women < 5.5 kg/m^2(3,19)^.

### Analysis of results and statistics

For data analysis, at first, we chose to describe predictor variables and outcomes, using absolute and relative frequencies. The normality of data was analyzed using the Kolmogorov-Smirnov test, using medians and interquartile amplitudes for age and educational level. After the variables were described, we analyzed the association between sociodemographic/clinical characteristics and the outcome “sarcopenia” using the chi-squared test, considering as significant associations where *p*<0.005.

For the multivariate analysis, all variables where *p* < 0.20 in the bivariate analysis were considered^(20)^. Furthermore, we applied the multinomial logistic regression model, since the distribution of the outcome has four categories^(21)^. This type of regression allows us to verify the association of each outcome category. In this research, the “no sarcopenia” category was adopted as a reference.

Furthermore, it is important to highlight that logistic regression results are presented as odds ratio (OR). Nonetheless, since this is a prevalence study, the delta method was used, which converts the OR to prevalence ratio (PR) of adjusted variables in the final model. Therefore, the effect size was found using PR, and the association strength was found using the confidence interval of 95% (CI95%). We considered associations where *p* < 0.05 were significant. All analyses were carried out using the software Stata 13.

## RESULTS

Sociodemographic variables showed a median age of 69 years (interquartile interval of 10), with the age group from 60 t0 79 years as predominant (87.5%; n = 336). Most participants were female (67.5%; n = 255), income of up to one minimum wage (55.5%; n = 213), retired (71.6%; n = 275), with no partner (65.4%; n = 251) and lived with family (77.3%; n = 297). The educational level median was 5 years (interquartile interval of 8), with 66.4 (n=255) having up to 8 years of study and 10.2% (n=39) illiterate.

Clinical data showed that 53.4% (n = 205) did not practice physical activity, 11.2% (n=43) drank, 9.1% (n=35) smoked, 47.7% (n=183) had fallen in the last 12 months. From these, 28.6% (n=110) had fallen twice or more in this period. Polypharmacy was present in 24.5% (n=94) of participants. According to the Body Mass Index (BMI) 25.2% (n=97) had low weight, 39.3% (n=44) normal weight, and 24% (n=92) were obese. Regarding their calf circumference, 12.8% (n=49) of participants had it below 31cm.

Regarding comorbidities, 64.8% (n = 249) had hypertension; 39.8%, (n = 153) diabetics; 23.4% (n = 80), osteoarthritis; 24.7% (n = 95), osteoporosis; 12.5% (n = 48), dyslipidemia; 10.5% (n = 39), cardiovascular disease; 4.9% (n = 19), cancer; 4.9% (n = 19), hypothyroidism; 3.6% (n = 14), anxiety; e 3.1% (n = 12), depression.

Table 1 shows the descriptive data related with the criteria established by the EWGSOP2 to evaluate sarcopenia.

**Table 1:** Variables that evaluate sarcopenia in elders attended in Primary Health Care Units (N = 384), Fortaleza, Ceará, Brazil, 2019

Variable		Category	n(%)	Mean (DP ±)	Min. - Max.
Grip Strength (dynamometer)	Female	< 16 kgf ≥ a 16 kgf	122 (31.8) 142 (37)	19.33 (±7.14)	2 - 46.5
	Male	< 27 kgf ≥ 27kgf	62 (16.1) 58 (15.1)		
Body Mass Index	Female	< 5.5 kg/m^2^ ≥ 5.5 kg/m^2^	126 (32.3) 138 (35.9)	6.46 (±1.39)	3.54 - 13.51
	Male	< 7 kg/m^2^ ≥ 7 kg/m^2^	19 (4.9) 101 (26.8)		
Gait Speed Test		**≤** 0.8 m/s	140 (36.5)	7.31s (±2.16)	3.98 - 28s
		> 0.8 m/s	244 (63.5)		

**Figure 1:**
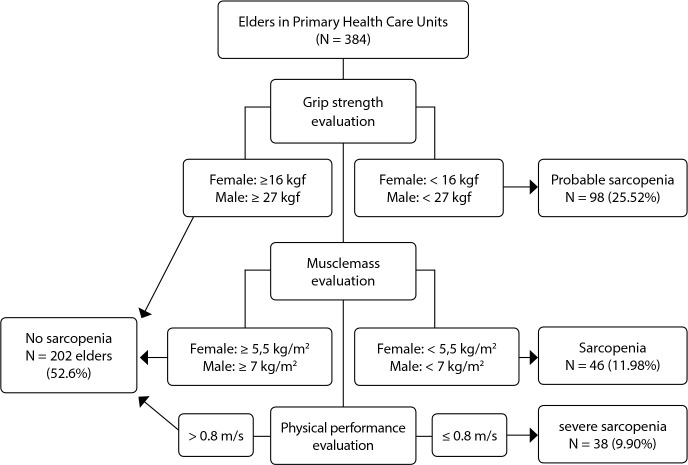
Criteria for the evaluation of the prevalence of sarcopenia in elders attended in the Primary Health Care Units (N=384), Fortaleza, Ceará, Brazil, 2019

Figure 1 shows the evaluation criteria and the prevalence of sarcopenia in elders registered in the PHCUs.

A bivariate analysis using sociodemographic and clinical data was carried out, with the possible outcomes no sarcopenia, probable sarcopenia, sociodemographic, and severe sarcopenia. Its main findings are presented in Table 2.

**Table 2:** Sociodemographic, clinical, and anthropometric characteristics of elders attended in Primary Health Care Units (N = 384), Fortaleza, Ceará, Brazil, 2019

Variables	SARCOPENIA				
	No sarcopenia (%)	Probable sarcopenia (%)	Sarcopenia (%)	Severe sarcopenia (%)	*p* ^*^
Age					0.377
80 years or older	24 (50.0)	10 (20.8)	6 (12.5)	8 (16.7)	
60 to 79 years	178 (53.0)	88 (26.2)	40 (11.9)	30 (8.9)	
Sex					0.005
Male	64 (51.2)	44 (35.2)	8 (6.4)	9 (7.2)	
Female	138 (53.3)	54 (20.8)	38 (14.7)	29 (11.2)	
Years of formal education					0.437
9 years or more	71 (55.0)	36 (27.9)	12 (9.3)	10 (7.7)	
Up to 8 years	131 (51.4)	62 (24.3)	34 (13.3)	28 (11.0)	
Marital Status					0.020
Has a partner	70 (52.6)	40 (30.1)	18 (13.5)	5 (3.8)	
Does not have a partner	132 (52.6)	58 (23.1)	28 (11.2)	33 (11.1)	
Physical exercise					0.128
Yes	100 (55.9)	38 (21.2)	26 (14.5)	15 (8.4)	
No	102 (49.8)	60 (29.2)	20 (9.8)	23 (11.2)	
Hypertension					0.187
Yes	121 (48.6)	70 (28.1)	31 (12.5)	27 (10.8)	
No	81 (60.0)	20 (20.7)	15 (11.1)	11 (8.2)	
Diabetes					0.049
Yes	68 (44.4)	48 (31.4)	22 (14.4)	15 (9.8)	
No	134 (58.0)	50 (21.6)	24 (10.4)	23 (10.0)	
Osteoarthritis					0.006
Yes	39 (43.3)	21 (23.3)	13 (14.5)	17 (18.9)	
No	163 (55.5)	77 (26.2)	33 (11.2)	21 (7.1)	
Osteoporosis					0.166
Yes	43 (45.3)	24 (25.3)	14 (14.7)	14 (174.7)	
No	159 (55.0)	74 (25.6)	32 (11.1)	24 (8.3)	
Dyslipidemia					0.012
Yes	23 (47.9)	7 (14.6)	12 (25.0)	6 (12.5)	
No	179 (53.3)	91 (27.1)	34 (10.1)	32 (9.5)	
Polypharmacy					< 0.001
Five or more medications	31 (33.0)	31 (33.0)	16 (17.0)	16 (17.0)	
Up to four medications	171 (59.0)	67 (23.1)	30 (10.3)	22 (7.6)	
Body Mass Index					< 0.001
Low weight	48 (49.5)	9 (9.3)	19 (19.6)	21 (21.6)	
Regular	80 (53.0)	35 (23.2)	22 (14.6)	14 (9.2)	
Overweight	22 (50.0)	17 (38.6)	9 (9.1)	1 (2.3)	
Obese	52 (56.5)	37 (40.2)	1 (1.1)	(2.2)	
Calf					< 0.001
< 31	14 (28.6)	8 (16.3)	13 (26.5)	14 (28.6)	
≥ 31	188 (56.1)	90 (26.9)	33 (9.8)	24 (7.2)	

* *Pearson's chi-squared test.*

The proportion of non-sarcopenic elders was similar between the sexes. Nonetheless, males showed 35.2% (n=44) of probable sarcopenia cases, 6.4% (n=8) of sarcopenia cases, and 7.2% (n=9) of severe sarcopenia cases (p=0.0005).

There was an equal distribution of elders with and without partners among non-sarcopenic elders, but the difference was larger among those with severe sarcopenia. The prevalence of severe sarcopenia was 11.1% (n=33) in those with no partners and 3.8% (n=5) in those with partners (p=0.020).

Most elders with some degree of sarcopenia received up to one minimum wage (p = 0.437), being: 27.7% (n = 59) of those with probable sarcopenia, 13.6% (n = 29) of those with sarcopenia, and 10.3% (n = 22), of those with severe sarcopenia. From those who lived alone, 16.5% (n=14) had probable sarcopenia; 11.8% (10) had sarcopenia; and 10.6% (n=9) had severe sarcopenia (p=0.435).

Regarding the number of falls, there was no association (p=0.202). The prevalence of elders who had fallen twice or more in the last year was 45.5% (n=50), among the non-sarcopenic, 31.8% (n=35) in the probable sarcopenic, 10.9% (n=12) in the sarcopenic, and 11.8% (n=13) in the severe sarcopenic.

Regarding drinking, its prevalence was higher in non-sarcopenic elders (67.4%; n=29), while 20.9% (n=9) had probable sarcopenia (p=0.171). The prevalence of smokers was also higher in the non-sarcopenic (45.7%, n=16), while 28.6% (n=10) had probable sarcopenia, 17.1% (n=6) sarcopenia, and 8.6% (n=3) had severe sarcopenia (p=0.701).

Regarding comorbidities, the prevalence of elders with osteoarthritis was higher among the sarcopenic (14.5%; n = 13) and severe sarcopenic (18.9%; n = 17) (p = 0.006). Similarly, the proportion of dyslipidemia was lower in the non-sarcopenic (47.9%; n = 23) and probable sarcopenic (14.6%; n = 7), while in the sarcopenic, the proportion was 25% (n = 12), and, in the severe sarcopenic, it was 12.5% (n = 6) (p = 0.012).

Polypharmacy was more frequent among the sarcopenic (33%; n = 31), the probable sarcopenic (17%; n = 16), and the severe sarcopenic (17%; n = 16) (p < 0.001).

Regarding BMI, all categories had a higher number of non-sarcopenic elders. Among low weight elders, most had severe sarcopenia (21,6%; n = 21) and sarcopenia (19.6%; n = 19); in elders with regular weight, most were probable sarcopenic (23.2%; n = 35) and sarcopenic (14.6%; n = 22). Among overweight and obese elders, most were probable sarcopenic: 38.6% (n=17) and 40.2% (n=37), respectively.

Finally, elders whose calf circumference was below 31 were more often the severe sarcopenic (28.6%; n = 14) and sarcopenic (26.5%; n = 13) (p < 0.001).

Table 3 shows the prevalence ratio of the sarcopenia outcome, after a multivariate analysis.

**Table 3:** Multivariate analysis of sociodemographic, clinical, and anthropometric characteristics associated with sociodemographic (N=384), Fortaleza, Ceará, Brazil, 2019

Variables	Sarcopenia ^*^PR (IC95%)					
	Probable sarcopenia	*p*	Sarcopenia	*p*	Severe sarcopenia	*p*
Sex (male)	1.75 (1.25 - 2.46)	0.001	0.50 (0.24 - 1.08)	0.077	1.14 (0.60 - 2.17)	0.679
Marital status (has a partner)	0.78 (0.56 - 1.09)	0.146	1.13 (0.63 - 2.01)	0.678	1.97 (0.88 - 4.38)	0.097
Physical exercise (yes)	1.05 (0.75 - 1.47)	0.796	1.53 (0.91 - 2.59)	0.110	0.39 (0.17 - 0.92)	0.031
Drinks (yes)	0.77 (0.56 - 1.08)	0.126	1.48 (0.87 - 2.51)	0.145	0.62 (0.35 - 1.09)	0.100
Hypertension (yes)	0.65 (0.36 - 1.18)	0.162	0.51 (0.13 - 1.93)	0.320	1.00 (0.39 - 2.56)	0.996
Diabetes (yes)	0.91 0.74 - 1.120	0.384	1.05 (0.72 - 1.55)	0.710	1.10 (0.60 - 2.01)	0.705
Osteoarthritis (yes)	1.39 (0.99 - 1.96)	0.060	0.96 (0.55 - 1.67)	0.873	0.70 (0.38 - 1.27)	0.241
Osteoporosis (yes)	0.96 (0.63 - 1.48)	0.869	1.02 (0.54 - 1.93)	0.948	2.16 (1.17 - 3.97)	0.013
Dyslipidemia (yes)	0.48 (0.24 - 0.98)	0.043	2.05 (1.13 - 3.73)	0.018	0.83 (0.37 - 1.86)	0.650
Polypharmacy (≥ 5 medications)	1.57 (1.10 - 2.56)	0.014	1.17 (0.62 - 2.22)	0.619	1.61 (0.86 - 3.05)	0.136
Body Mass Index						
Low weight	0.46 (0.24 - 0.90)	0.023	1.34 (0.76 - 2.38)	0.312	1.66 (0.89 - 3.10)	0.109
Regular	1	-	1	-	1	-
Overweight	1.78 (1.20 - 2.61)	0.003	0.91 (0.37 - 2.32)	0.832	0.35 (0.06 - 2.02)	0.242
Obesity	1.72 (1.19 - 2.50)	0.003	0.12 (0.02 - 0.88)	0.037	0.29 (0.08 - 1.09)	0.067
Calf (< 31)	0.98 (0.53 - 1.80)	0.936	2.24 (1.23 - 4.07)	0.009	2.19 (1.16 - 4.17)	0.016

* *Prevalence ratio and association strength using a 95% confidence interval.*

Males are 1.75 times (CI95%: 1.25 - 2.46) more likely to be probable sarcopenic as opposed to non-sarcopenic. Regarding the other outcomes, there was no difference regarding gender. When it comes to physical activities, elders who practice some form of exercise had a lower prevalence of severe sarcopenia (PR: 0.39; CI95%:0.17 - 0.92). Sarcopenic elders were 2.05 times (CI95%: 1.13-3.73) more likely to show dyslipidemia. Osteoporosis was 2.16 times (CI95%:1.17-3.97) more prevalent in those with severe sarcopenia. Polypharmacy, in turn, increased the prevalence of probable sarcopenia in 1.57 (1.10-2.56).

Regarding BMI, the prevalence of overweight elders was 1.78 times (CI95%: 1.20-2.61) higher in those with probable sarcopenia. Finally, obesity was 1.72 (CI95%:1.19-2.50) times more prevalent in elders with probable sarcopenia. Finally, calf circumferences lower than 31 cm increased the prevalence of sarcopenia in 2.24 times (CI95%:1.23 - 4.07) and the prevalence of severe sarcopenia in 2.19 times (CI95%:1.16 - 4.17).

## DISCUSSION

The prevalence of probable sarcopenia, sarcopenia, and severe sarcopenia are similar to that found in other Brazilian cities and in international studies^(22-24)^, albeit the lack of a single classification system makes it harder to find more robust information^(5)^. Finding a consensual diagnosis would facilitate not only research, but also the discovery of treatment options and the translation of the investigation results into practice^(10)^.

To do so, professionals must be able to incorporate evaluation actions to be able to determine adequate interventions^(25)^. The methods used in this study can be used by primary health care workers - especially by the nurse, due to their relevant role in identifying the attention needs of individuals in primary care, as well as in health promotion and protection^(26)^.

In this study, males were more likely to develop probable sarcopenia. Further studies also found higher prevalence and risk for sarcopenia in men^(27-28)^. Still, in general, literature states that being female is the risk factor for sarcopenia^(29)^, considering that, starting with 50 years, the loss of strength in women is hastened due to hormonal changes in their non-reproductive stages^(30)^.

Nonetheless, primary care is directed towards children and women. This feminine environment causes feelings of invulnerability in men, which in turn leads them to seek mostly emergency services and specialized consultations when they lose functionality^(31-32)^. That said, the findings of this study open space for new research, specifically with male elders.

It was found that dyslipidemia is associated with probable sarcopenia and sarcopenia. Studies show associations between dyslipidemia and the development of sarcopenia^(33-34)^. Nonetheless, pathologic mechanisms are still unknown. It has been suggested that higher fat levels cause inflammatory cytokines, such as tumor necrosis factor alpha and interleukins, to be secreted, reducing muscle tissue^(33-34)^.

The association of physical exercise and severe sarcopenia should be remarked upon. Studies show that, the less physical activity, the less the muscle mass, and the greater the prevalence of physical disabilities^(35-36)^. The regular practice of exercise delays muscle loss and increases muscle strength, preventing sarcopenia. Literature shows that the best results are achieved with Progressive Resisted Exercise (PRE)^(35-36)^; furthermore, when the exercise is supervised by professionals, it is more beneficial to treat sarcopenia, improving muscle mass, strength, and physical performance when compared to exercise carried out at home unsupervised^(37)^.

Regarding comorbidities, research shows the association between osteoporosis and the development of sarcopenia^(38)^. It stands out that sarcopenia and osteoporosis have the same etiology (inflammation, hormonal and nutritional deficiency, and lack of physical exercise) and the same risk factors for muscle incapacity^(39)^. Also, osteoarthritis limits mobility due to pain and rigidity, reducing muscle strength.

Regarding the presence of diabetes, there was no association, in disagreement with the results from other studies^(28,40)^. Nonetheless, it is necessary to study this element in more depth in future investigations, since endocrine changes and the liberation of inflammatory cytokines in diabetes lead to muscle degradation. Additionally, insulin resistance is a multifactorial condition, and aging and obesity are associated with a chronic inflammatory state that causes skeletal muscle loss, being this muscle the main target-tissue responsive to insulin, contributing for sarcopenia^(41)^.

In this study, polypharmacy was the most prevalent characteristic for elders with any degree of sarcopenia, corroborating international studies^(42-43)^. The use of several medications is common in aging and increases the risk of adverse reactions. These can interfere in the metabolism and homeostasis, causing mitochondrial dysfunction and hydroelectric and endocrine unbalances, as well as gastrointestinal absorption dysfunctions, all of which are factors that lead to the development of sarcopenia^(42-43)^.

In regard to findings involving body composition, Lee’s equation method, which considers BMI and calf circumference, is considered to be an effective method to evaluate weight reduction and sarcopenia^(44)^. Body composition may hide sarcopenia obesity, characterized by high levels of body fat, which catalyzes the reduction of lean body mass and muscle force^(45-46)^. It was worth saying that identifying sarcopenic obesity is difficult for health workers^(46)^. Still, measuring muscle mass is a challenge to be implemented in practice due to the limitations intrinsic to evaluation instruments (such as cost, availability, and ease of use), which are often more useful for research than useful in clinical practice, especially for Primary Care^(47)^.

Furthermore, although European Consensus is advertised as a valid way to evaluate sarcopenia, it has limitations in the evaluation of elders with physical and cognitive restrictions. As a result, the perception about the actual prevalence of sarcopenia is mistaken^(48)^. Bedridden and wheelchair bound elders are more likely to develop comorbidities and, thus, to lose functionality^(48-49)^. This corroborates the need for research to develop diagnostic methods and strategies capable of including elders with physical and cognitive restrictions.

Considering the above, we must note that studies of this nature generate important information that can serve as guide for managers and professionals to plan and develop interventions for people of specific ages. Sarcopenia is a topic that has become increasingly relevant and requires studies that address new treatment options and preventive interventions.

### Study limitations

Limitations include the fact that some independent variables (educational level and comorbidities) were self-reported, making it impossible to establish cause relations due to the study design. Furthermore, it was not possible to evaluate sarcopenia in elderly with cognitive restrictions, and in those who were bedridden or could not go to the units.

Also, we suggest longitudinal studies to be carried out in order to evaluate in depth the development of sarcopenia in the elderly population, both in urban and rural areas.

### Contributions to the Field of Nursing

The proposal of evaluating the prevalence and characteristics of sarcopenia in primary care will make it possible to replicate the study in other settings. Furthermore, we expect encouraging nurses to carry out further studies on the topic, which is still seldom discussed by nursing workers.

In addition, this study stands out due to its originality, and the information generated will give support for interventions for the prevention and promotion of the health of the elder.

## CONCLUSIONS

In the elders attended in the Primary Health Care at Fortaleza, the prevalence of probable sarcopenia was 25.52%; sarcopenia, 11.8%; and severe sarcopenia, 9.90%. We suggest using the same methods used in this study for evaluations in primary care.

Sex, osteoporosis, polypharmacy, overweight, obesity, and calf circumference below 31 cm are the most present characteristics of some degree of sarcopenia, while physical activity is less prevalent among those with severe sarcopenia. Some of these characteristics are modifiable conditions; therefore, systematic evaluations, in addition to lifestyle changes, could prevent the installation and the repercussions of sarcopenia on the quality of life of elders and their families.
